# Simplification of Dietary Treatment in Pharmacoresistant Epilepsy: Impact of C8 and C10 Fatty Acids on Sirtuins of Neuronal Cells In Vitro

**DOI:** 10.3390/nu16111678

**Published:** 2024-05-29

**Authors:** Miriam Rebekka Rühling, Hans Hartmann, Anibh Martin Das

**Affiliations:** Clinic for Pediatric Kidney-, Liver- and Metabolic Diseases, Hannover Medical School, Carl-Neuberg-Str. 1, 30625 Hannover, Germany; ruehling.miriam@mh-hannover.de (M.R.R.); hartmann.hans@mh-hannover.de (H.H.)

**Keywords:** ketogenic diet, sirtuins, medium-chain fatty acids, ß-hydroxybutyrate, pharmacoresistant epilepsy

## Abstract

Pharmacotherapy is the therapeutic mainstay in epilepsy; however, in about 30% of patients, epileptic seizures are drug-resistant. A ketogenic diet (KD) is an alternative therapeutic option. The mechanisms underlying the anti-seizure effect of a KD are not fully understood. Epileptic seizures lead to an increased energy demand of neurons. An improvement in energy provisions may have a protective effect. C8 and C10 fatty acids have been previously shown to activate mitochondrial function in vitro. This could involve sirtuins (SIRTs) as regulatory elements of energy metabolism. The aim of the present study was to investigate whether ß-hydroxybutyrate (ßHB), C8 fatty acids, C10 fatty acids, or a combination of C8 and C10 (250/250 µM) fatty acids, which all increase under a KD, could up-regulate SIRT1, -3, -4, and -5 in HT22 hippocampal murine neurons in vitro. Cells were incubated for 1 week in the presence of these metabolites. The sirtuins were measured at the enzyme (fluorometrically), protein (Western blot), and gene expression (PCR) levels. In hippocampal cells, the C8, C10, and C8 and C10 incubations led to increases in the sirtuin levels, which were not inferior to a ßHB incubation as the ‘gold standard’. This may indicate that both C8 and C10 fatty acids are important for the antiepileptic effect of a KD. A KD may be replaced by nutritional supplements of C8 and C10 fatty acids, which could facilitate the diet.

## 1. Introduction

Epilepsy has a worldwide prevalence of approximately 1% [1]. In the paediatric age group, 500/100,000 children are affected [2]. Epilepsy is related to neuronal death, apoptosis, oxidative stress, and mitochondrial dysfunction [3].

A ketogenic diet (KD)—a high-fat diet with the severe restriction of carbohydrates [1,4]—has been shown to be effective in paediatric patients with pharmacoresistant epilepsy [5]. A KD led to a 50% seizure reduction in 60% of patients with pharmacoresistant epilepsy [1]. However, a KD poses a considerable burden to affected patients and their families in terms of palatability, gastrointestinal side effects, and adherence, often resulting in the premature cessation of the diet [6]. Resolving these unmet needs by modifying the diet would be an asset. 

Several forms of KDs have been suggested, such as the classical KD with a fixed lipid-to-carbohydrate ratio, the Atkins diet with a defined carbohydrate intake, and the MCT (medium-chain triglyceride) diet [7]. The MCT diet allows for a greater carbohydrate intake and does not require calculations because it is based on a defined number of calories from MCT oil to produce ketones [8]. All these forms of KDs lead to an increased production of ketone bodies in the liver [9]. However, they are quite demanding; a simplified dietary intervention would, therefore, be helpful. 

Ketone bodies cross the blood–brain barrier and are utilized by the brain. They are converted to acetyl-coenzyme A and can thus enter the citric acid cycle so that they are used for energy production [10]. 

A KD can increase the function of the mitochondrial respiratory chain and ATP production. In addition, oxidative stress can be reduced [1]. However, the exact mechanism of how KDs exert these effects is not fully understood [10].

C8 (octanoic acid or caprylic acid) and C10 (decanoic acid or capric acid) fatty acids, which increase under a KD [11], have been shown to activate mitochondrial energy metabolism and increase mitochondrial biogenesis in vitro [12], which may be explained by the up-regulation of sirtuins [13]. 

In addition, C10 can reduce the release of excitatory neurotransmitters and the network excitability in vitro, which plays an important role in epilepsy [14]. Furthermore, C10 is known to increase the seizure threshold in mice as well as the mitochondrial number and the respiratory chain complex and catalase activities [14].

Epileptic seizures lead to an increased energy demand of neurons with subsequent neuronal damage and an unstable membrane potential [15,16], which may increase the likelihood of further seizures [17]. Furthermore, a plethora of other effects have been ascribed to single sirtuins, as summarized by Guarente [18,19] and Mishra [3]. 

The observations that C8 and C10 compounds activate cellular energy metabolism [12] prompted the development of commercial nutritional supplements containing C8 and C10 fatty acids (Keto Epi^®^ and KVita^®^), which can be added to low-glycaemic-index (LGI) nutrition to mimic a high-fat, low-carbohydrate diet. In a previous study in the UK, KVita^®^ was shown to have an antiepileptic effect in vivo, though this study had limitations (heterogeneous patient group) [14]. 

We hypothesized that the activation of energy metabolism by C8 and C10 fatty acids is mediated by an up-regulation of sirtuins. In the present study, we examined the combined effect of C8 and C10 fatty acids (comparable to the nutritional supplements described above) on SIRT1, -3, and -5 at the enzyme and expression levels in murine hippocampal neurons. SIRT4 could only be analysed at the gene expression level. Classically, it was supposed that beta-hydroxybutyrate (ßHB) exclusively mediates the antiepileptic effect of a KD. Therefore, we incubated cells with ßHB (as the ‘gold standard’) in parallel to the incubations with C8 and/or C10 fatty acids and examined the impact on sirtuins.

C8 and C10 fatty acids may have beneficial effects beyond epilepsy in disorders where a compromised mitochondrial function is supposed to play a pathophysiological role, such as Parkinson’s disease, cancer, neurodegenerative diseases, and inborn errors of mitochondrial energy metabolism [13].

## 2. Materials and Methods

### 2.1. Cell Culture

For this study, HT22 murine hippocampal neuronal cells from an immortalized murine cell line were used (kindly provided by Prof. K. Haastert-Tallini, Institute of Neuroanatomy, Hannover Medical School). This cell model was used because epilepsy often originates from hippocampal cells and patients with pharmacoresistant epilepsy often have alterations in the hippocampus [20]. 

The cells were cultured in T25 culture flasks (Sarstedt AG, Nümbrecht, Germany) at 37 °C and 5% CO_2_ in Dulbecco’s modified Eagle medium (DMEM) (Thermo Scientific, Waltham, MA, USA) supplemented with 10% (*v*/*v*) foetal bovine serum (Biowest SAS, Nuaillé, France) and 1% (*v*/*v*) penicillin/streptomycin (Sigma-Aldrich, Steinheim, Germany). 

To mimic a ketogenic/LGI environment, we added only 1 g of glucose per litre to the medium, which was devoid of glucose. Usually, the recommended standard concentration of glucose in the culture medium is 4.5 g per litre for HT22 cells. Lower concentrations than 1 g of glucose per litre prevented cell proliferation and induced cell death.

The cells were incubated for 1 week in the presence of different metabolites, and the media were changed every other day. The cells were separated into six different groups with different supplements. The cells were either incubated without supplements (controls) or sham-incubated with ethanol (concentration in the medium of 0.5%), which served as the carrier of medium-chain fatty acids, with C8 (250 µM), C10 (250 µM), C8 and C10 in combination (50% and 50%, respectively, as in KetoEpi^®^, which resulted in concentrations of 250 µM C8 and C10, respectively), or ß-hydroxybutyrate (5 mM). 

The concentrations of the C8 and C10 metabolites used in the in vitro cell study were the concentrations that could be detected in the cerebrospinal fluid (CSF) of mice and rats undergoing a ketogenic diet therapy [21,22]. Another article showed that 5 mM ßHB is the recommended concentration for ketogenic diet experiments, because 5 mM ßHB is the usual concentration reached in the plasma of patients undergoing ketogenic diet therapy [23].

The cell cultures were incubated in duplicate, and 4 incubation runs were performed for each incubation condition. 

After one week of incubation, the cells were harvested. For the Western blot analysis, the cells were lysed using the Laemmli buffer; to determine the sirtuin enzyme activity, we used the HEPES buffer; and for RNA isolation, we used the RLT buffer (Qiagen, Hilden, Germany). 

### 2.2. Analytical Procedures

#### 2.2.1. Sirtuin Enzyme Activity

The enzyme activities of SIRT1, -3, and -5 were analysed using the fluorescence-based enzyme activity kit (Enzo Life Sciences, Lausen, Switzerland) from Enzo Life Sciences GmbH, as detailed elsewhere [11]. Specific substrates for SIRT1 (FLUOR DE LYS^®^ SIRT1 Substrate), SIRT3 (FLUOR DE LYS^®^ SIRT3 Substrate), and SIRT5 (FLUOR DE LYS^®^ SIRT5 Substrate) (Enzo Life Sciences, Lausen, Switzerland) were used. The substrates, assay buffer, and developer were provided with the test kit. A 96 (half-area)-well plate was used; for the enzyme activity of SIRT1 and SIRT3, a transparent plate was used, and for the enzyme activity of SIRT5, a black plate was used. On this plate, the SIRT1, -3, and -5 standards and the probes were pipetted. All the samples were pipetted and measured in triplicate. The samples for the measurements of SIRT1 and SIRT3 were diluted to 1:5 with the Hepes buffer. The samples for the quantification of the SIRT5 activity were not diluted. For the blanks in the standard series, only the assay buffer was used. The special substrates were added to the wells with the samples and the standard series. The plates were subsequently incubated at 37 °C for 15 min, followed by the addition of the developer reagent (consisting of 76% assay buffer, 20% ‘Developer Reagent 2’ and 4% 50 mM nicotinamide (NAM)) to the samples and the standard series to stop the enzyme reaction. Subsequently, the plate was incubated for 45 min at 37 °C. The enzyme activities were measured using the Tecan© fluorescence plate reader (Tecan group Ltd., Männedorf, Switzerland) (emission at 360 nm and detection at 460 nm). To calculate the enzyme activity, Microsoft Excel (Microsoft Office LTSC Professional Plus 2021) was used. No measurements of SIRT4 were possible due to the absence of an adequate fluorescence-based enzyme activity kit.

#### 2.2.2. Bicinchoninic Assay (BCA)

To measure the protein concentration in the probes, a Pierce BCA protein assay kit (Thermo Scientific, Waltham, MA, USA) was used. A full-area 96-well plate was used, and the albumin standard (provided with the assay) and the probes (diluted to 1:3 using ddH₂O) were pipetted in duplicate into the 96-well plate. A mixture of reagent A and reagent B (both provided by the Pierce BCA protein assay kit (Thermo Scientific, USA)) was pipetted onto the probes and the standard for development. The plate was incubated for 60 min at 60 °C. The colorimetric reaction was detected at a wavelength of 562 nm using a Tecan© 96-well plate reader (Tecan group Ltd., Switzerland). 

#### 2.2.3. Protein Expression

To analyse the protein expression of the different sirtuins, a semi-dry Western blotting technique was used, as described elsewhere [10]. An equal number of cells (5000 cells/µL) were lysed after harvesting in the Laemmli buffer. For electrophoresis, SDS-PAGE gels (10% separating gel and 4% collecting gel) were used, and for the transfer, a nitrocellulose membrane was used. A total of 10 µL per sample was applied to the gel pockets after heating at 95 °C for 15 min, followed by centrifugation at 10,000 rpm. The electrophoresis was carried out in a Bio-Rad electrophoresis chamber using a running buffer. The transfer to the nitrocellulose membrane was performed at a constant current of 200 mA for 20 min. After the membrane was washed, the Intercept blocking buffer (TBS) was used for blocking. Overnight, the membranes were incubated with the primary antibodies. Rabbit anti-SIRT1 (Millipore, Darmstadt, Germany), goat anti-SIRT3 (Abcam, Cambridge, UK), rabbit anti-SIRT5 (Millipore, Germany), and rabbit anti-beta-actin (Cell Signaling Technology, Inc., Denvers, CO, USA) primary antibodies were used. Thereafter, the membranes were washed and then incubated with a secondary antibody. Secondary anti-rabbit and anti-goat antibodies were used (Li-cor Biosciences, Lincoln, NE, USA). Beta-actin was used to normalize the protein expression of sirtuins in the respective probes. Detection was performed with the Odyssey FC system (Li-cor biosciences, USA). No measurements of SIRT4 were possible at the protein level due to the absence of adequate antibodies.

#### 2.2.4. Gene Expression

RNA isolation and cDNA synthesis were carried out by using the RNeasy mini kit and the Omniscript RT kit, respectively, which were provided by Quiagen, Venlo, Germany, as described elsewhere [10]. SIRT5 could not be analysed at the gene expression level due to the absence of adequate primers. A 96-well plate was used. Into the plate, 4.2 µL of cDNA and 5.8 µL of master mix (5 µL of SYBR green + 0.8 µL of primer (forward to reverse) were pipetted per well. Murine primers for SIRT1, -3, and -4 and housekeeper genes (B2M, HPRT1, and ACT-B) were used for the PCR. The SIRT1, -3, and -4 gene expressions were measured using SYBR green-based real-time polymerase chain reaction (PCR). Beta-2-microglobulin (B2M), hypoxanthine phosphoribosyltransferase 1 (HPRT1), and beta-actin (ACT-B) were used as internal controls, and the relative sirtuin gene expression was calculated based on a normalization method using the three internal controls. 

#### 2.2.5. Statistical Analysis

The results were statistically evaluated and analysed using SAS Enterprise Guide 7.1. An analysis of variance was used as a test procedure. The results are shown as the mean + SD, and *p* < 0.05 was regarded as significant.

## 3. Results

### 3.1. Sirtuin 1 (SIRT1)

#### 3.1.1. Impact of Different Metabolites on SIRT1 Enzyme Activity

**Figure 1:**
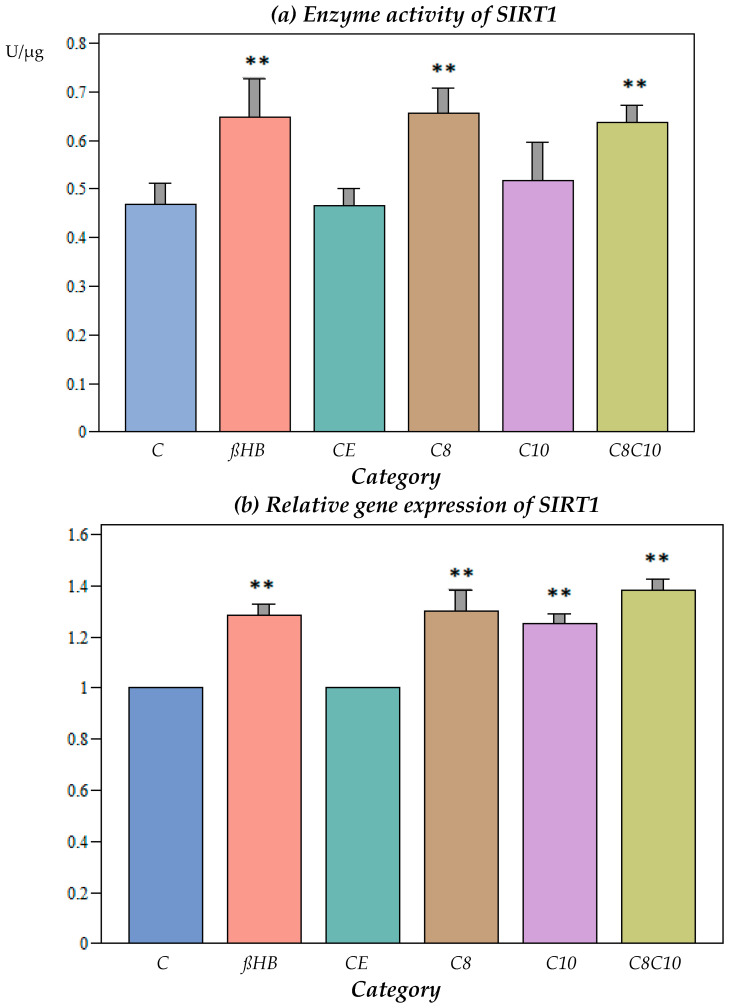

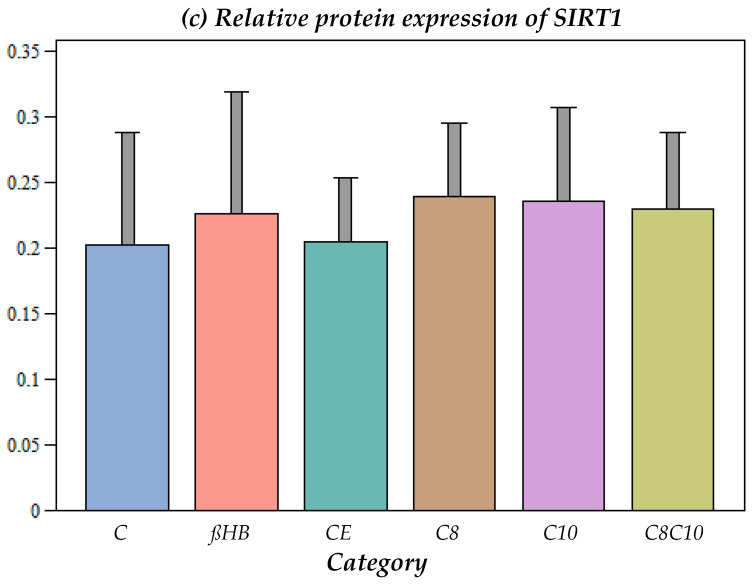
(**a**) Enzyme activity of SIRT1 under different incubation conditions. C: control, ßHB: ß-hydroxybutyrate, CE: ethanol control, C8: C8 fatty acid (octanoic acid), C10: C10 fatty acid (decanoic acid), C8C10: C8 and C10 fatty acids in combination. Mean + SD; n: 8; ** *p* < 0.01 vs. control. (**b**) Gene expression of SIRT1 under different incubation conditions. C: control, ßHB: ß-hydroxybutyrate, CE: ethanol control, C8: C8 fatty acid (octanoic acid), C10: C10 fatty acid (decanoic acid), C8C10: C8 and C10 fatty acids in combination. Mean + SD; n: 8; ** *p* < 0.01 vs. control. (**c**) Protein content (Western blot) of SIRT1 under different incubation conditions. C: control, ßHB: ß-hydroxybutyrate, CE: ethanol control, C8: C8 fatty acid (octanoic acid), C10: C10 fatty acid (decanoic acid), C8C10: C8 and C10 fatty acids in combination. Mean + SD, n: 8.

Ethanol, which was used as a solvent for the medium-chain fatty acids, did not have an effect on the SIRT1 activity (Figure 1a). 

The incubation of the cells with C8 fatty acids resulted in a significant increase in the SIRT1 activity compared to the control, while the C10 fatty acids did not result in any alterations to the enzyme activity. The combined incubation with the C8 and C10 compounds resulted in a significant increase in the SIRT1 activity, but did not lead to further enzyme activation compared to incubation with C8 alone. 

ßHB resulted in a significant increase in the SIRT1 activity compared to the control, but there was no significant difference when compared to the C8 or the combined C8 and C10 metabolites (Figure 1a).

#### 3.1.2. Impact of Different Metabolites on Gene Expression of SIRT1

At the gene expression level, SIRT1 increased significantly when the cells were incubated with C8, C10, C8 and C10, or ßHB (Figure 1b).

#### 3.1.3. Impact of Different Metabolites on Protein Content of SIRT1

No significant differences could be observed for protein expression under the different incubation conditions compared to the controls (Figure 1c).

#### 3.1.4. Correlation Analysis between Gene Expression and Enzyme Activity for SIRT1

There was a significant positive correlation between the enzyme activity and gene expression for SIRT1. A correlation coefficient of 0.3965 (*p* < 0.001) was observed.

### 3.2. Sirtuin 3 (SIRT3)

#### 3.2.1. Impact of Different Metabolites on SIRT3 Enzyme Activity

**Figure 2:**
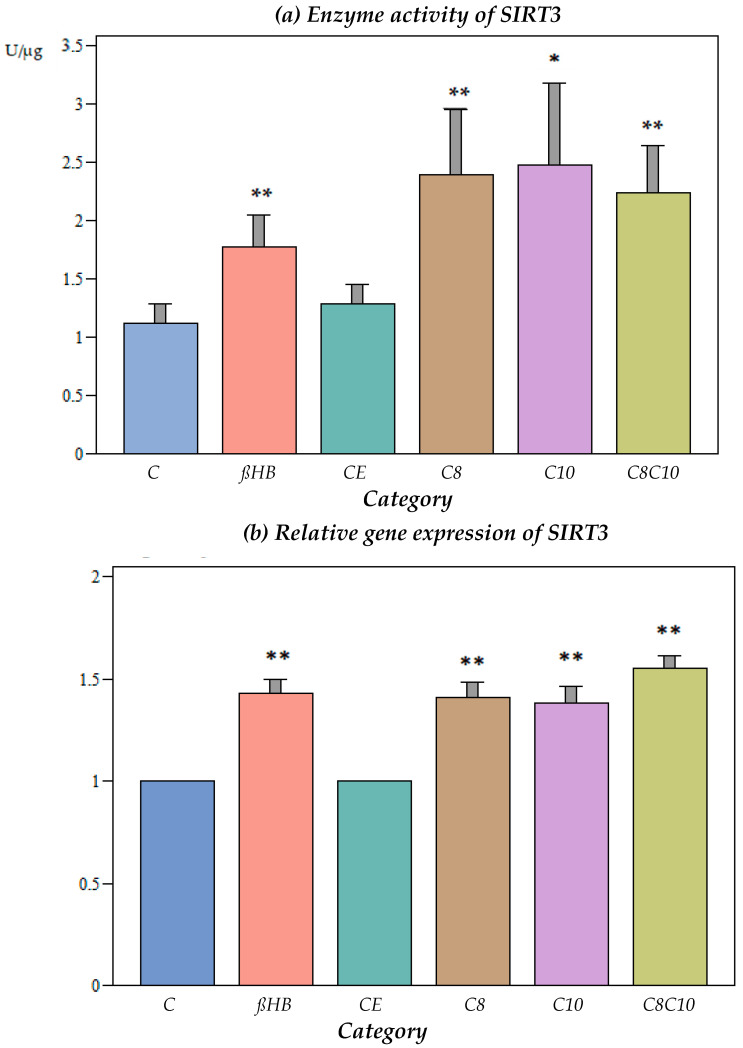

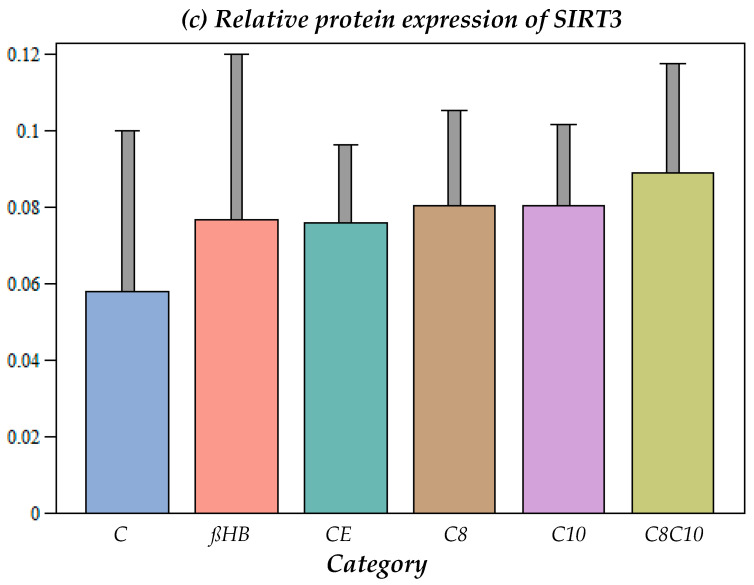
(**a**) Enzyme activity of SIRT3 under different incubation conditions. C: control, ßHB: ß-hydroxybutyrate, CE: ethanol control, C8: C8 fatty acid (octanoic acid), C10: C10 fatty acid (decanoic acid), C8C10: C8 and C10 fatty acids in combination. Mean + SD; n: 8; * *p* < 0.05; ** *p* < 0.01 vs. control. (**b**) Gene expression of SIRT3 under different incubation conditions. C: control, ßHB: ß-hydroxybutyrate, CE: ethanol control, C8: C8 fatty acid (octanoic acid), C10: C10 fatty acid (decanoic acid), C8C10: C8 and C10 fatty acids in combination. Mean + SD; n: 8; ** *p* < 0.01 vs. control. (**c**) Protein content (Western blot) of SIRT3 under different incubation conditions. C: control, ßHB: ß-hydroxybutyrate, CE: ethanol control, C8: C8 fatty acid (octanoic acid), C10: C10 fatty acid (decanoic acid), C8C10: C8 and C10 fatty acids in combination. Mean + SD, n: 8.

Ethanol, which was used as a vehicle for the medium-chain fatty acids, did not have an effect on the SIRT3 activity (Figure 2a). 

The incubation of the cells with ßHB or C8, C10, or a combination of C8 and C10 fatty acids resulted in a significant increase in the SIRT3 enzyme activity compared to the controls. The activation of SIRT3 activity was less pronounced with ßHB compared to the fatty acids.

#### 3.2.2. Impact of Different Metabolites on Gene Expression of SIRT3

At the gene expression level, SIRT3 increased significantly when the cells were incubated with C8, C10, C8 and C10, or ßHB (Figure 2b).

#### 3.2.3. Impact of Different Metabolites on Protein Content of SIRT3

No significant differences were found under the different incubation conditions compared to the controls (Figure 2c).

#### 3.2.4. Correlation Analysis between Gene Expression and Enzyme Activity for SIRT3

There was a significant positive correlation between the enzyme activity and gene expression for SIRT3. The correlation coefficient was 0.3154 (*p* < 0.05).

### 3.3. Sirtuin 4 (SIRT4)

#### Impact of Different Metabolites on Gene Expression of SIRT4

**Figure 3:**
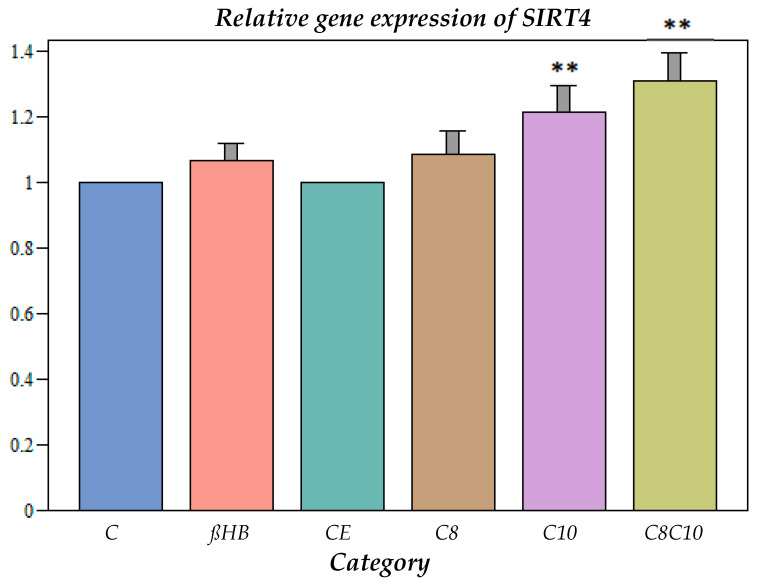
Gene expression of SIRT4 under different incubation conditions. C: control, ßHB: ß-hydroxybutyrate, CE: ethanol control, C8: C8 fatty acid (octanoic acid), C10: C10 fatty acid (decanoic acid), C8C10: C8 and C10 fatty acids in combination. Mean + SD; n: 8; ** *p* < 0.01 vs. control.

The incubation with C10 fatty acids as well as a combination of C8 and C10 compounds resulted in a significant increase in the gene expression (Figure 3).

### 3.4. Sirtuin 5 (SIRT5)

#### 3.4.1. Impact of Different Metabolites on SIRT5 Enzyme Activity

**Figure 4:**
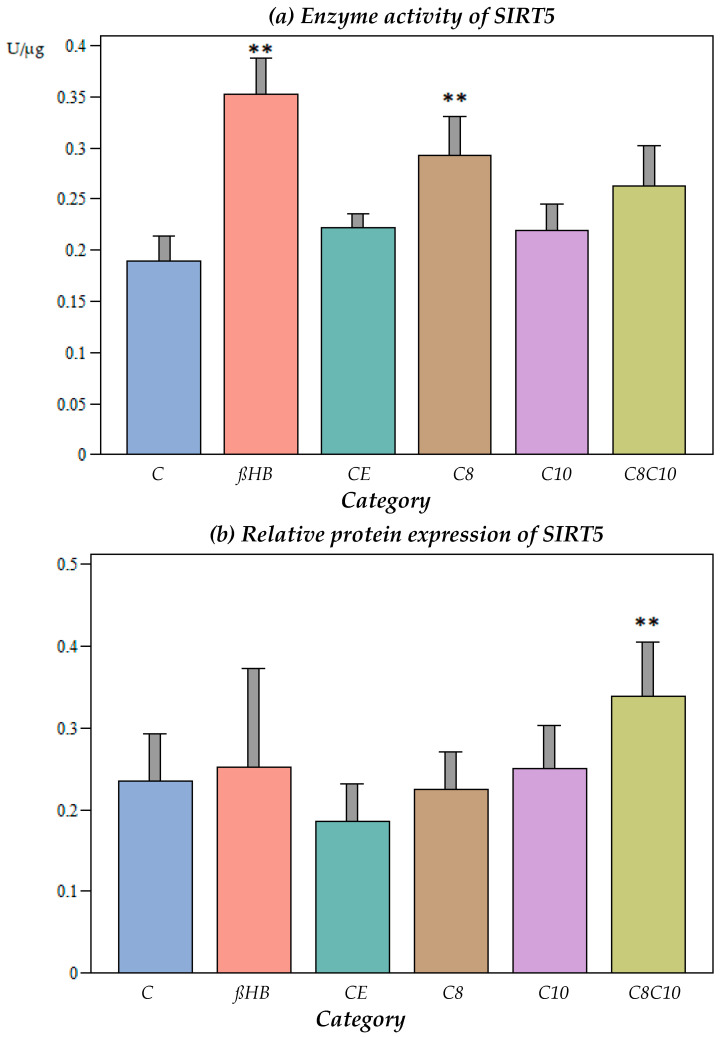
(**a**) Enzyme activity of SIRT5 under different incubation conditions. C: control, ßHB: ß-hydroxybutyrate, CE: ethanol control, C8: C8 fatty acid (octanoic acid), C10: C10 fatty acid (decanoic acid), C8C10: C8 and C10 fatty acids in combination. Mean + SD; n: 8; ** *p* < 0.01 vs. control. (**b**) Protein content (Western blot) of SIRT5 under different incubation conditions. C: control, ßHB: ß-hydroxybutyrate, CE: ethanol control, C8: C8 fatty acid (octanoic acid), C10: C10 fatty acid (decanoic acid), C8C10: C8 and C10 fatty acids in combination. Mean + SD; n: 8; ** *p* < 0.01 vs. control.

Ethanol, which was used as a vehicle for the medium-chain fatty acids, did not have an effect on the SIRT5 activity (Figure 4a). 

The incubation of the cells with C8 fatty acids or ßHB resulted in a significant increase in the SIRT5 activity compared to the control (*p* < 0.05), while the C10 fatty acids did not result in any alterations to the enzyme activity (*p* > 0.05). Interestingly, the combined incubation with C8 and C10 compounds did not lead to further enzyme activation compared to C8 alone. 

#### 3.4.2. Impact of Different Metabolites on Protein Content of SIRT5

At the protein level, no significant differences were found under the different incubation conditions compared to the controls, with the exception of the combined C8 and C10 fatty acid incubation, which resulted in an increase in the SIRT5 protein content (Figure 4b).

## 4. Discussion

Clinically, the use of a KD is known to be successful in many patients with pharmacoresistant epilepsy. However, its mechanism of action has not been elucidated yet. We hypothesized that sirtuins, as regulatory elements of biochemical and physiological pathways, are up-regulated under a KD. KDs are known to elevate not only ßHB, the predominant ketone body, but also medium-chain fatty acids such as decanoic acid (C10) and octanoic acid (C8). 

This prompted us to study the impact of ßHB and the C8 and C10 fatty acids on sirtuins. If the antiepileptic effect is mediated by one or several of these compounds, single substances may be used to mediate the antiepileptic effect. This may be less challenging than following a full KD. 

Oral ßHB is not palatable and is frequently associated with gastrointestinal side effects, resulting in poor adherence [24]. Medium-chain fatty acids are better tolerated. Therefore, decanoic acid and octanoic acid have been suggested as oral alternatives to a KD. The biochemical rationale for this is the positive impact of medium-chain fatty acids on energy metabolism in vitro [12], presumably preventing epileptic seizures and neuronal damage [3]. Sirtuins, as regulators of energy metabolism and other biochemical functions, may mediate the effect of C10 and C8 fatty acids. We therefore compared the impact of decanoic acid and octanoic acid on sirtuins, with the effect of ßHB used as the ‘gold standard’.

### 4.1. Sirtuin 1 (SIRT1)

Incubation with C8, C8 and C10, or ßHB resulted in significant increases in the SIRT1 activity (Figure 1b). This was reflected in the gene expression, where significant increases were observed under all the incubation conditions (Figure 1b). However, this did not result in elevated protein expression for SIRT1 (Figure 1c). As the sirtuin activity increased under metabolite incubations, a posttranslational modification by the metabolites C8, C10, and ßHB was postulated. Sirtuin 1 is mainly found in the cell nucleus, but it has also been detected in the cytoplasm and mitochondria [25,26,27] and could, therefore, have a direct posttranslational effect on mitochondrial metabolism.

SIRT1 is supposed to play a crucial role in the pathophysiology of epileptic seizures [3]. Interestingly, it was previously shown that the down-regulation of SIRT1 in the rat hippocampus following status epilepticus led to decreased mitochondrial respiratory chain activity, impaired mitochondrial biogenesis, and higher oxidative stress [28]. Therefore, the activation of energy metabolism by the induction of sirtuins in pharmacoresistant epilepsy seems to be an attractive protective concept.

SIRT1, as a nicotinamide adenine dinucleotide (NAD^+^)-dependent histone deacetylase, is important and crucial for metabolic health by deacetylating many target proteins in numerous tissues, including the brain [28,29,30,31,32]. Furthermore, important systemic effects are indirectly regulated by SIRT1 via the hypothalamus [19]. 

The SIRT1 activity in the brain plays an essential role in both mitochondrial function and ROS detoxification. The activation of SIRT1—as observed in this study under different incubation conditions—can result in increased mitochondrial function and biogenesis; improved energy homeostasis; and reduced inflammation, oxidative stress, cell apoptosis, and autophagy [3,33]. Furthermore, oxidative stress is reduced by the activation of SIRT1 [3,34,35], which is mediated by PGC1-alpha (proliferator-activated receptor gamma coactivator 1 alpha) and PARP (poly-ADP-ribose polymerase) [3]. 

Furthermore, SIRT1 plays a crucial role in DNA damage repair processes [36], and can thus protect neurons.

The beneficial effects of SIRT1 in terms of the mitochondrial energy supply and repair processes have given rise to the assumption that the up-regulation of SIRT1 by medium-chain fatty acids can play an important role in the treatment of drug-resistant epilepsies [3,37,38]. These effects by medium-chain fatty acids are not inferior to those of ßHB.

### 4.2. Sirtuin 3 (SIRT3)

Similar to SIRT1, SIRT3 was up-regulated at the enzyme level by ßHB and by C8, C10, and the combination of C8 and C10 fatty acids, which was reflected at the gene expression level, but not at the protein level. Thus, increased SIRT3 activity cannot be explained by a higher protein level of the enzyme. Therefore, posttranslational changes by the metabolites at the enzyme level were postulated.

As with SIRT1, SIRT3 has been shown to play a crucial role in metabolic processes. Both sirtuins are activated by elevated NAD(+) levels, which is the result of a decreased energy content within the cells, especially in the mitochondrial compartment. Both SIRT1 and SIRT3 deacetylate various proteins and increase the cellular energy balance [31].

SIRT3 is localized in the mitochondria, and thus, can have a direct posttranslational effect on mitochondrial proteins. As described in a recent review [39], SIRT3 is highly expressed in the brain. SIRT3 has a positive effect on energy homeostasis, the respiratory chain complexes, mitochondrial quality control, and the redox balance [39,40]; hence, its up-regulation by C8 and C10 fatty acids could have a positive impact on the neuronal energy balance, which may mediate the protective effect of these compounds in drug-refractory epilepsy [31].

In addition to respiratory chain complexes, enzymes that are involved in fatty acid oxidation, the tricarboxylic acid cycle, and redox homeostasis are also deacetylated by SIRT3 [40,41,42,43,44], further improving the energy balance.

### 4.3. Sirtuin 4 (SIRT4)

SIRT4 could only be measured at the level of gene expression, where a significant increase compared to the controls occurred after incubation with all metabolites. If this up-regulation resulted in increased enzyme activities for SIRT4, this could have implications for mitochondrial fatty acid oxidation (ß-oxidation). SIRT4 inhibits the breakdown of malonyl-coenzyme A, which reduces the influx of long-chain fatty acids into the mitochondria, so that less fatty acid oxidation takes place and fewer ketone bodies are formed in the liver. The increased gene expression of SIRT4, therefore, may lead to reduced fatty acid oxidation [23].

Previously, it was believed that neurons cannot metabolize fatty acids. In recent years, it has been shown that neurons can produce ketone bodies from fatty acids and use fatty acids to produce energy via ß-oxidation [45]. In another study, it was observed that astrocytes in brain slices from mice can use C8 and C10 fatty acids for energy production and the synthesis of GABA [46].

SIRT4 is also predominantly localized in the mitochondria, and deacetylates and inactivates malonyl CoA decarboxylase (MCD). This enzyme regulates the relationship between lipid synthetic processes and fatty acid oxidation. MCD inhibition by elevated SIRT4 levels—as observed in our study—will result in elevated malonyl CoA levels and inhibit fatty acid oxidation [47]. This may result in increased lipid synthesis with an altered cellular lipid composition, as observed in a previous study under C10-fatty-acid incubation [10].

Furthermore, SIRT4 has been shown to be neuroprotective by reducing oxidative stress and maintaining the glutamate concentration in the neuronal synapse [48].

### 4.4. Sirtuin 5 (SIRT5)

The SIRT5 enzyme activity was only significantly up-regulated in the presence of ßHB or C8 fatty acids, but not C10 or the combination of C8 and C10 fatty acids. The protein levels of SIRT5 were only elevated when the cells were incubated with C8 and C10 fatty acids in combination.

SIRT5 is also predominantly localized in the mitochondria and is supposed to have an antioxidative function by expressing superoxide dismutase 2 (SOD2), thus protecting neuronal cells [49,50]. SIRT5 plays a role in the regulation of the urea cycle by modulating the carbamoylphosphate synthase 1 (CPS1) activity, and thus, it has an influence on the ammonia levels [51].

The effect of a combination of octanoic acid and decanoic acid on the enzyme activities of SIRT1, -3, and -4 was not inferior to that of ßHB as the ‘gold standard’. However, the impact of the combination of fatty acids on SIRT5 was less pronounced compared to ßHB. Interestingly, C8 incubation alone had a similar effect as ßHB. Apart from exerting an antioxidant effect, SIRT5 is involved in the regulation of protein substrates that are involved in glycolysis, the TCA cycle, fatty acid oxidation, the electron transport chain, ketone body formation, and ROS detoxification, among others, and it is also involved in the regulation of the urea cycle, which is not found in the brain [23]. The extracerebral effects of SIRT5 dysfunction may have an effect on the brain. However, this has to be clarified in future in vivo studies.

A KD is not only a therapeutic option in pharmacoresistant epilepsy, but is advocated in certain inborn errors of metabolism, such as a pyruvate dehydrogenase deficiency, a glucose transporter 1 (GLUT1) deficiency, hyperinsulinism, respiratory chain defects, and others [52]. Apart from respiratory chain defects, where C8 and C10 fatty acids can lead to an up-regulation of mitochondrial function, therapy with medium-chain fatty acids is presumably not an option in these conditions, as the biochemical rationale is the use of ketone bodies as an alternative energy substrate. 

## 5. Conclusions

In cultured murine hippocampal cells, C8, C10, and C8 and C10 fatty acid incubations led to increases in the levels of different sirtuins, which were not inferior to ßHB incubation. This probably results in an improved energy supply and reduced oxidative stress in the brain during epileptic seizures, which has a neuroprotective effect. Therefore, both C8 and C10 are important for the antiepileptic effect and are not inferior to incubation with ßHB. A KD may be replaced by nutritional supplements of C8 and C10 fatty acids, which could facilitate the diet and enhance compliance. 

In future in vitro studies, we intend to measure the impact of octanoic acid and decanoic acid as well as ßHB on the mitochondrial respiratory chain complexes.

To translate these in vitro findings to clinical medicine, a follow-up study in vivo is planned in which children suffering from pharmacoresistant epilepsy will receive commercially available C8 and C10 supplements while following an LGIT. This may enhance compliance. We intend to measure the sirtuins in the blood of the treated patients.

## Data Availability

The original contributions presented in the study are included in the article, further inquiries can be directed to the corresponding author.
